# Examining the association between anthropometric parameters and telomere length and mortality risk

**DOI:** 10.18632/oncotarget.15976

**Published:** 2017-03-07

**Authors:** Chen-Jung Wu, Tung-Wei Kao, Yuan-Yung Lin, Fang-Yih Liaw, Li-Wei Wu, Yaw-Wen Chang, Tao-Chun Peng, Wei-Liang Chen

**Affiliations:** ^1^ Department of Family and Community Medicine, Division of Family Medicine, Tri-Service General Hospital and School of Medicine, National Defense Medical Center, Taipei, Taiwan, Republic of China; ^2^ Department of Community Medicine, Division of Family Medicine, Taoyuan Armed Forces General Hospital, Taoyuan, Taiwan, Republic of China; ^3^ Department of Family and Community Medicine, Division of Geriatric Medicine, Tri-Service General Hospital and School of Medicine, National Defense Medical Center, Taipei, Taiwan, Republic of China; ^4^ Graduate Institute of Clinical Medical, College of Medicine, National Taiwan University, Taipei, Taiwan, Republic of China; ^5^ Department of Otolaryngology–Head and Neck Surgery, Tri-Service General Hospital, and School of Medicine, National Defense Medical Center, Taipei, Taiwan, Republic of China; ^6^ Graduate Institute of Medical Sciences, National Defense Medical Center, Taipei, Taiwan, Republic of China

**Keywords:** anthropometric indices, telomere length, mortality risk, Gerotarget

## Abstract

A shorter telomere length is associated with several systemic disorders. Telomere length may be an informative biomarker for the maintenance of the overall health status and mortality. There are a limited number of empirical studies concerning the effect of anthropometric parameters on telomere length. The data are derived from the National Health and Nutrition Examination Survey from 1999 to 2002. The primary outcomes of this study were to examine the potential relationships between the anthropometric indices and the telomere length, while secondary outcomes of this study was to investigate the association between different anthropometric indices and mortality risk. A significant positive correlation was noted between the mean telomere length and the thigh circumference (TC) and calf circumference (CC) in all designed models. Participants in the highest TC and CC quartiles tended to have a longer telomere length and lowered the hazards for all-cause mortality to 43% and 57%, respectively. Notably, the anthropometric indices involving the CC with higher values seemed to be surrogate markers for the reduction of the risk of all-cause, cardiovascular and malignancy-related mortality (all *P* < 0.05). The CCmay be a valuable tool to guide public health policy and a clinical prognostic indicator for the risk of mortality.

## INTRODUCTION

Telomeres belong to the protective nucleoprotein structures that cap the ends of eukaryotic chromosomes with an uncomplicated tandemly repeated telomeric DNA sequence. Generally, end nucleotides are naturally lost and telomeres shorten with the aging process via mitosis during cell division [1,2]. Some age-related diseases have been regarded as a group of short telomere defects, such as idiopathic pulmonary fibrosis, dyskeratosis congenita, and bone marrow failure [3]. The manifestations of these unique genetic diseases are referred to as “telomere syndromes” [4]. The possible cumulative effect of the shortening of the telomere length was reported in many earlier studies. One review article demonstrated the close relevance of a shorter telomere length with several systemic disorders, including type 2 diabetes mellitus, cardiovascular disease, stroke and the risk of cancers such as bladder, esophageal, gastric, and renal cancers [5]. Furthermore, a recent study indicated that telomere shortening was a marker of sleep disturbances, cognitive impairment and senile dementia [6]. Telomere length may be an informative biomarker for the maintenance of the overall health status in elderly populations [7]. Emerging evidence has suggested that a positive connection between mortality and a shorter telomere length. One prospective cohort study involving 47,102 participants in 2013 demonstrated that a short telomere length had a positive association with an increased possibility of early death after cancer [8]. Rode and his colleagues pointed out that a clear dose-response association between telomere length and mortality in the general population [9]. Additionally, another prospective cohort study with follow-up for a median of 10.2 years indicated that a shorter telomer length significantly increased the risk of all-cause mortality in patients with type 2 diabetes mellitus [10]. These results implied that genetically determined a short telomere length was associated with high risk of all-cause mortality.

The factors associated with telomere length shortening are conflicting with the exception of aging. Accumulating evidence indicated an association between pertinent risk factors and a short telomere length, including a high body mass index (BMI), higher total and abdominal adiposity [11], regular consumption of sugar-sweetened beverages [12], inappropriate healthy behavior, a low education level [13], severity of obstructive sleep apnea syndrome [14], and a higher allostatic load, which is often regarded as a type of biological effect in response to cumulative and chronic stress related to subsequent adverse health outcomes [15]. Notably, an increasing number of recent publications had shown that running-specific physical activity and higher cardiorespiratory fitness had positive associations with relatively a longer telomere length or recent telomere length attrition [16, 17]. Another prospective cohort study published in 2016 addressed that weight loss induced by bariatric surgery contributed to a significant increase in the telomere length, which was different from telomere attrition [18].

Anthropometric parameters other than BMI provided unique prognostic information concerning subcutaneous fat, muscle mass and fat accumulation, including the arm circumference (AC), waist circumference (WC), thigh circumference (TC) and calf circumference (CC). A substantial body of research had documented that the WC had negative associations with cardiovascular and all-cause mortality [19]. Apart from the WC, there was little research concerning the effects of other anthropometric parameters on telomere length and specific-cause mortality. Because there was insufficient research evidence to expound on these relationships, we focused on the interplay among the anthropometric indices, telomere length, and mortality risk.

## RESULTS

### Preliminary analysis

The results of the association analysis between the mean telomere length and different anthropometric parameters (AC, WC, TC and CC) obtained using three regression models are listed in Table 1. A significant positive correlation was noted between the mean telomere length and the TC and CC, whereas a significant negative correlation was observed between the mean telomere length and the WC in all designed models (*P* < 0.05). The unadjusted β coefficient of the telomere length for the TC and CC was 0.004 (95% confidence intervals (CI), 0.003-0.005, *P* < 0.001 and 0.002-0.005, *P* < 0.001, respectively). Notably, the additional adjustment of all covariates did not change the significance in our study. Taken together, these findings demonstrated that the subjects with higher TC and CC or lower WC tended to have a longer relative telomere length.

**Table 1:** Association between the telomere length and the anthropometric parameters

Anthropometric Parameters	Model ^a^ 1 β^b^ (95% CI)	*P* Value	Model ^a^ 2 β^b^ (95% CI)	*P* Value	Model ^a^ 3 β^b^ (95% CI)	*P* Value
**AC (cm)**	−0.001 (−0.002, 0.001)	0.316	0.002 (−0.001, 0.006)	0.113	0.002 (−0.001, 0.006)	0.108
**WC (cm)**	−0.002 (−0.003, 0.002)	<0.001	−0.001 (−0.002, 0.000)	0.006	−0.001 (−0.002, 0.000)	0.008
**TC (cm)**	0.004 (0.003, 0.005)	<0.001	0.004 (0.002, 0.006)	<0.001	0.004 (0.002, 0.006)	<0.001
**CC (cm)**	0.004 (0.002, 0.005)	<0.001	0.003 (0.001, 0.006)	0.018	0.003 (0.001, 0.006)	0.019

^a^ Adjusted covariates:

Model 1 = Unadjusted

Model 2 = Model 1 + age, sex, race/ethnicity, BMI, systolic blood pressure, serum fasting glucose, serum cholesterol, serum albumin, ALT, serum uric acid, C-reactive protein

Model 3 = Model 2 + history of congestive heart failure, coronary heart disease, angina/angina pectoris, heart attack, stroke, cancer/malignancy, smoking, moderate to vigorous recreational activity

^b^ β coefficients was interpreted as change of telomere length for each increase in different anthropometric parameters

Abbreviation:

AC, arm circumference; WC, waist circumference; TC, thigh circumference; CC, calf circumference; BMI, body mass index; ALT, alanine aminotransferase

### Study sample characteristics

Based on the positive association of the TC and CC with the telomere length, we divided the anthropometric parameters into quartiles in eligible subjects. Table 2 demonstrates the characteristics of all subjects classified TC quartiles; a total of 48.3% of the participants were men and the mean age was 49.08 ± 18.64 years. A higher telomere length, younger age and lower prevalence of heart attack, coronary heart disease, congestive heart failure, stroke and malignancy were significantly prominent in the higher TC quartiles compared with the lowest quartile. The clinical characteristics of the study population divided by CC quartiles are summarized in Table 3 with similar results. A total of 48.0% of the participants were men and the mean age was 49.25 ± 18.72 years. A higher telomere length and younger age were still present, but a significantly lower prevalence was discovered in congestive heart failure and malignancy.

**Table 2:** Characteristics of study participants of quartiles of thigh circumference

	Quartiles of Thigh Circumference					
Characteristics of Study Participants	Q1 (<48.1)	Q2 (48.1 to<52.1)	Q3 (52.1 to<56.7)	Q4 (>56.7)	Total	*P*
	(*n* = 1899)	(*n* = 1869)	(*n* = 1869)	(*n* = 1870)	(*n* = 7507)	Value
**Continuous variables^a^**						
**Mean telomere length** **(T/S ratio)**	0.98 (0.26)	1.04 (0.33)	1.04 (0.26)	1.07 (0.27)	1.03 (0.28)	<0.001
**Age (years)**	56.84 (20.42)	49.30 (18.63)	46.78 (16.75)	43.25 (15.63)	49.08 (18.64)	<0.001
**BMI (kg/m**^2^)	23.01 (3.22)	26.06 (3.10)	28.79 (3.40)	35.11 (5.87)	28.25 (6.03)	<0.001
**SBP (mm-Hg)**	131.89 (26.63)	125.84 (21.66)	125.47 (19.51)	125.29 (18.43)	127.13 (21.97)	<0.001
**Serum FG (mg/dL)**	98.73 (41.48)	96.87 (35.27)	95.41 (28.42)	97.57 (33.64)	97.15 (35.05)	0.031
**Serum TC (mg/dL)**	200.11 (41.53)	200.69 (42.44)	202.20 (41.52)	200.04 (39.88)	200.76 (41.35)	0.349
**Serum albumin (g/dL)**	4.33 (0.36)	4.36 (0.35)	4.32 (0.36)	4.23 (0.35)	4.31 (0.36)	<0.001
**ALT (U/L)**	22.88 (47.02)	25.77 (32.73)	27.47 (21.75)	27.78 (25.73)	25.96 (33.34)	<0.001
**Serum uric acid (mg/dL)**	5.03 (1.49)	5.19 (1.46)	5.50 (1.51)	5.65 (1.51)	5.34 (1.51)	<0.001
**Serum CRP (mg/dL)**	0.45 (1.21)	0.40 (0.77)	0.42 (0.63)	0.64 (0.87)	0.48 (0.90)	<0.001
**Categorical variables^b^**						
**Male**	819 (43.1)	955 (51.1)	1041 (55.7)	810 (43.3)	3625 (48.3)	<0.001
**Non-Hispanic white**	1008 (53.1)	950 (50.8)	979 (52.4)	882 (47.2)	3819 (50.9)	<0.001
**Congestive heart failure**	71 (3.7)	53 (2.8)	36 (1.9)	46 (2.5)	206 (2.7)	<0.001
**Coronary heart disease**	110 (5.8)	78 (4.2)	71 (3.8)	49 (2.6)	308 (4.1)	<0.001
**Angina/angina pectoris**	87 (4.6)	66 (3.5)	51 (2.7)	58 (3.1)	262 (3.5)	0.091
**Heart attack**	109 (5.7)	84 (4.5)	62 (3.3)	52 (2.8)	307 (4.1)	<0.001
**Stroke**	74 (3.9)	50 (2.7)	44 (2.4)	41 (2.2)	209 (2.8)	0.008
**Cancer or malignancy**	224 (11.8)	155 (8.3)	117 (6.3)	113 (6.0)	609 (8.1)	<0.001
**Smoking**	976 (51.4)	903 (48.4)	934 (50.0)	829 (44.3)	3642 (48.5)	0.002
**Moderate to vigorous** **recreational activity**	883 (46.5)	848 (45.4)	830 (44.4)	818 (43.7)	3379 (45.0)	0.350

BMI, body mass index; SBP, systolic blood pressure; Serum FG, serum fasting glucose; Serum TC, serum total cholesterol; ALT, alanine aminotransferase; Serum CRP, serum C-reactive protein

^a^ Values were expressed as mean (standard deviation)

^b^ Values in the categorical variables were expressed as number (%)

**Table 3:** Characteristics of study participants of quartiles of calf circumference

	Quartiles of Calf Circumference					
Characteristics of Study Participants	Q1 (<35.3)	Q2 (35.3 to<37.8)	Q3 (37.8 to<40.6)	Q4 (>40.6)	Total	*P*
	(*n* = 1961)	(*n* = 1914)	(*n* = 1864)	(*n* = 1890)	(*n* = 7629)	Value
**Continuous variables^a^**						
**Mean telomere length** **(T/S ratio)**	1.01 (0.28)	1.03 (0.32)	1.04 (0.26)	1.05 (0.26)	1.03 (0.28)	<0.001
**Age (years)**	53.29 (21.25)	50.00 (19.01)	48.19 (17.39)	45.32 (15.89)	49.25 (18.72)	<0.001
**BMI (kg/m**^2^)	23.22 (3.47)	26.30 (3.43)	28.87 (3.77)	34.95 (6.11)	28.31 (6.12)	<0.001
**SBP (mm-Hg)**	129.86 (26.26)	127.02 (22.85)	126.07 (20.00)	125.98 (17.92)	127.25 (22.07)	<0.001
**Serum FG (mg/dL)**	97.14 (39.40)	96.66 (35.47)	96.82 (31.89)	98.33 (33.57)	97.24 (35.24)	0.454
**Serum TC (mg/dL)**	199.99 (42.43)	201.11 (41.96)	202.09 (42.05)	200.14 (39.31)	200.82 (41.47)	0.371
**Serum albumin (g/dL)**	4.31 (0.38)	4.34 (0.35)	4.32 (0.36)	4.25 (0.35)	4.31 (0.36)	<0.001
**ALT (U/L)**	23.59 (52.93)	25.26 (22.71)	26.70 (25.32)	28.10 (18.91)	25.88 (33.12)	<0.001
**Serum uric acid (mg/dL)**	4.95 (1.52)	5.22 (1.45)	5.47 (1.46)	5.76 (1.51)	5.34 (1.52)	<0.001
**Serum CRP (mg/dL)**	0.45 (1.09)	0.43 (0.86)	0.47 (0.85)	0.60 (0.82)	0.49 (0.92)	<0.001
**Categorical variables^b^**						
**Male**	763 (38.9)	921 (48.1)	1024 (54.9)	956 (50.6)	3664 (48.0)	<0.001
**Non-Hispanic white**	887 (45.2)	935 (48.9)	983 (52.7)	1060 (56.1)	3865 (50.7)	<0.001
**Congestive heart failure**	72 (3.7)	50 (2.6)	50 (2.7)	41 (2.2)	213 (2.8)	0.035
**Coronary heart disease**	96 (4.9)	74 (3.9)	75 (4.0)	68 (3.6)	313 (4.1)	0.066
**Angina/angina pectoris**	70 (3.6)	67 (3.5)	69 (3.7)	62 (3.3)	268 (3.5)	0.901
**Heart attack**	104 (5.3)	72 (3.8)	76 (4.1)	68 (3.6)	320 (4.2)	0.090
**Stroke**	78 (4.0)	52 (2.7)	45 (2.4)	47 (2.5)	222 (2.9)	0.061
**Cancer or malignancy**	203 (10.4)	151 (7.9)	133 (7.1)	135 (7.1)	622 (8.2)	0.002
**Smoking**	979 (49.9)	920 (48.1)	907 (48.7)	890 (47.1)	3696 (48.5)	0.150
**Moderate to vigorous** **recreational activity**	895 (45.6)	862 (45.0)	860 (46.1)	817 (43.2)	3434 (45.0)	0.296

BMI, body mass index; SBP, systolic blood pressure; Serum FG, serum fasting glucose; Serum TC, serum total cholesterol; ALT, alanine aminotransferase; Serum CRP, serum C-reactive protein

^a^ Values were expressed as mean (standard deviation)

^b^ Values in the categorical variables were expressed as number (%)

### Association between the telomere length and anthropometric parameters

To determine the relationship between the telomere length and the anthropometric parameters, we performed a multivariate analysis to clarify the effect of the TC and CC. Table 4 shows a positive association in the highest TC and CC quartiles with telomere length in all models (*P* < 0.05). Participants in the higher TC and CC quartiles tended to have significantly higher telomere lengths (*P* for trend < 0.05). These findings showed a dose-response association between telomere length and TC and CC.

**Table 4:** Association between the thigh/calf circumference and the telomere length

Models ^a^	Quartiles	β^b^ (95% CI)	*P* Value	*P* for Trend	β^b^ (95% CI)	*P* Value	*P* for Trend
Anthropometric Parameters		Thigh Circumference			Calf Circumference		
**Model 1**	Q2 v.s. Q1 Q3 v.s. Q1 Q4 v.s. Q1	0.050 (0.031, 0.069) 0.048 (0.029, 0.067) 0.083 (0.064, 0.102)	<0.001 <0.001 <0.001	<0.001	0.016 (−0.003, 0.034) 0.024 (0.005, 0.043) 0.040 (0.021, 0.059)	0.105 0.012 <0.001	<0.001
**Model 2**	Q2 v.s. Q1 Q3 v.s. Q1 Q4 v.s. Q1	0.024 (0.005, 0.043) 0.019 (−0.002, 0.040) 0.045 (0.016, 0.074)	0.012 0.080 0.002	0.011	0.011 (−0.007, 0.029) 0.017 (−0.003, 0.037) 0.030 (0.004, 0.056)	0.220 0.095 0.025	0.027
**Model 3**	Q2 v.s. Q1 Q3 v.s. Q1 Q4 v.s. Q1	0.024 (0.005, 0.042) 0.018 (−0.003, 0.039) 0.044 (0.015, 0.073)	0.014 0.088 0.003	0.013	0.011 (−0.007, 0.029) 0.017 (−0.003, 0.037) 0.030 (0.004, 0.056)	0.231 0.101 0.024	0.026

^a^ Adjusted covariates:

Model 1 = Unadjusted

Model 2 = Model 1 + age, sex, race/ethnicity, BMI, systolic blood pressure, serum fasting glucose, serum cholesterol, serum albumin, ALT, serum uric acid, C-reactive protein

Model 3 = Model 2 + history of congestive heart failure, coronary heart disease, angina/angina pectoris, heart attack, stroke, cancer/malignancy, smoking, moderate to vigorous recreational activity

^b^ β coefficients was interpreted as change of telomere length for each increase in different anthropometric parameters

Abbreviation: BMI, body mass index; SBP, systolic blood pressure; ALT, alanine aminotransferase

### Hazard ratios of all-cause mortality categorized by the different anthropometric indices

To investigate the association between different anthropometric indices and mortality risk, Table 5 demonstrates the hazard ratios (HRs) of all-cause mortality in subjects with different anthropometric indices. The HRs and 95% CIs of the highest quartile compared with the base quartile for individuals with TC, CC, TC/WC, CC/WC and (TC + CC/WC) domains in the unadjusted model were 0.221 (95% CI: 0.163 to 0.298), 0.250 (95% CI: 0.186 to 0.337), 0.084 (95% CI: 0.055 to 0.128), 0.124 (95% CI: 0.086 to 0.179), and 0.090 (95% CI: 0.060 to 0.136), respectively (all *P* < 0.001). The HRs and 95% CIs of the highest quartile compared with the base quartile in the totally adjusted model of the different anthropometric indices listed above were 0.569 (95% CI: 0.350 to 0.924; *P* = 0.023), 0.426 (95% CI: 0.280 to 0.649; *P* < 0.001), 0.491 (95% CI: 0.307 to 0.785; *P* = 0.003), 0.431 (95% CI: 0.286 to 0.650; *P* < 0.001) and 0.430 (95% CI: 0.271 to 0.685; *P* < 0.001), respectively. Collectively, the findings provided that all of the anthropometric indices investigated in our study had an inverse correlation with all-cause mortality, that had higher values significantly lowered the hazard (especially CC, which showed the lowest hazard ratio).

**Table 5:** HRs of all-cause mortality categorized by the anthropometric parameters

Anthropometric Parameters	Quartiles	Unadjusted Model HR (95% CI)	*P* Value	Total Adjusted Model HR (95% CI)	*P* Value
**TC**	Q2 v.s. Q1 Q3 v.s. Q1 Q4 v.s. Q1	0.433 (0.343,0.547) 0.276 (0.210, 0.362) 0.221 (0.163, 0.298)	<0.001 <0.001 <0.001	0.669 (0.515, 0.868) 0.512 (0.364, 0.723) 0.569 (0.350, 0.924)	0.002 <0.001 0.023
**CC**	Q2 v.s. Q1 Q3 v.s. Q1 Q4 v.s. Q1	0.496 (0.394, 0.624) 0.366 (0.284, 0.472) 0.250 (0.186, 0.337)	<0.001 <0.001 <0.001	0.598 (0.467, 0.766) 0.497 (0.367, 0.673) 0.426 (0.280, 0.649)	<0.001 <0.001 <0.001
**TC/WC**	Q2 v.s. Q1 Q3 v.s. Q1 Q4 v.s. Q1	0.351 (0.278, 0.443) 0.200 (0.150, 0.247) 0.084 (0.055, 0.128)	<0.001 <0.001 <0.001	0.680 (0.533, 0.869) 0.704 (0.510, 0.974) 0.491 (0.307, 0.785)	0.002 0.034 0.003
**CC/WC**	Q2 v.s. Q1 Q3 v.s. Q1 Q4 v.s. Q1	0.397 (0.315, 0.501) 0.278 (0.214, 0.361) 0.124 (0.086, 0.179)	<0.001 <0.001 <0.001	0.566 (0.444, 0.721) 0.652 (0.488, 0.871) 0.431 (0.286, 0.650)	<0.001 0.004 <0.001
**(TC + CC)/WC**	Q2 v.s. Q1 Q3 v.s. Q1 Q4 v.s. Q1	0.382 (0.304, 0.481) 0.216 (0.163, 0.287) 0.090 (0.060, 0.136)	<0.001 <0.001 <0.001	0.658 (0.517, 0.838) 0.657 (0.480, 0.901) 0.430 (0.271, 0.685)	0.001 0.009 <0.001

Adjustment for age, sex, race/ethnicity, BMI, SBP, serum fasting glucose, serum cholesterol, serum albumin, ALT, serum uric acid, C-reactive protein, history of congestive heart failure, coronary heart disease, angina/angina pectoris, heart attack, stroke, cancer/malignancy, smoking, moderate to vigorous recreational activity

Abbreviation:BMI, body mass index; SBP, systolic blood pressure; ALT, alanine aminotransferase; WC, waist circumference; TC, thigh circumference; CC, calf circumference

### Hazard ratios of cardiovascular mortality categorized by the different anthropometric indices

Table 6 demonstrates the HRs of cardiovascular mortality in the subjects with different anthropometric indices. The HRs and 95% CIs of the highest quartile compared with the base quartile for individuals with TC, CC, TC/WC, CC/WC and (TC + CC/WC) domains in the unadjusted model were 0.168 (95% CI: 0.098 to 0.285), 0.168 (95% CI: 0.095 to 0.297), 0.026 (95% CI: 0.008 to 0.082), 0.068 (95% CI: 0.032 to 0.147), and 0.018 (95% CI: 0.004 to 0.073), respectively (all *P* < 0.001). The HRs and 95% CIs of the highest quartile compared with the base quartile in the totally adjusted model in the different anthropometric indices listed above were 0.525 (95% CI: 0.224 to 1.233; *P* = 0.139), 0.407 (95% CI: 0.189 to 0.877; *P* = 0.022), 0.205 (95% CI: 0.062 to 0.678; *P* = 0.009), 0.297 (95% CI: 0.130 to 0.678; *P* = 0.004) and 0.114 (95% CI: 0.027 to 0.482; *P* = 0.003), respectively. These findings showed higher CC and CC/WC levels were significantly associated with lower cardiovascular mortality in all quartiles.

**Table 6:** HRs of cardiovascular mortality categorized by the anthropometric parameters

Anthropometric Parameters	Quartiles	Unadjusted Model HR (95% CI)	*P* Value	Total Adjusted Model HR (95% CI)	*P* Value
**TC**	Q2 v.s. Q1 Q3 v.s. Q1 Q4 v.s. Q1	0.315 (0.209, 0.477) 0.153 (0.089, 0.265) 0.168 (0.098, 0.285)	<0.001 <0.001 <0.001	0.538 (0.338, 0.856) 0.324 (0.168, 0.627) 0.525 (0.224, 1.233)	0.009 0.001 0.139
**CC**	Q2 v.s. Q1 Q3 v.s. Q1 Q4 v.s. Q1	0.430 (0.290, 0.637) 0.318 (0.206, 0.493) 0.168 (0.095, 0.297)	<0.001 <0.001 <0.001	0.608 (0.397, 0.933) 0.539 (0.318, 0.912) 0.407 (0.189, 0.877)	0.023 0.021 0.022
**TC/WC**	Q2 v.s. Q1 Q3 v.s. Q1 Q4 v.s. Q1	0.246 (0.161, 0.376) 0.142 (0.084, 0.241) 0.026 (0.008, 0.082)	<0.001 <0.001 <0.001	0.525 (0.337, 0.818) 0.671 (0.373, 1.206) 0.205 (0.062, 0.678)	0.004 0.182 0.009
**CC/WC**	Q2 v.s. Q1 Q3 v.s. Q1 Q4 v.s. Q1	0.340 (0.228, 0.506) 0.200 (0.123, 0.325) 0.068 (0.032, 0.147)	<0.001 <0.001 <0.001	0.547 (0.361, 0.830) 0.567 (0.335, 0.960) 0.297 (0.130, 0.678)	0.005 0.035 0.004
**(TC + CC)/WC**	Q2 v.s. Q1 Q3 v.s. Q1 Q4 v.s. Q1	0.288 (0.191, 0.434) 0.176 (0.107, 0.288) 0.018 (0.004, 0.073)	<0.001 <0.001 <0.001	0.545 (0.355, 0.836) 0.638 (0.370, 1.098) 0.114 (0.027, 0.482)	0.006 0.105 0.003

Adjustment for age, sex, race/ethnicity, BMI, SBP, serum fasting glucose, serum cholesterol, serum albumin, ALT, serum uric acid, C-reactive protein, history of congestive heart failure, coronary heart disease, angina/angina pectoris, heart attack, stroke, cancer/malignancy, smoking, moderate to vigorous recreational activity

Abbreviation: BMI, body mass index; SBP, systolic blood pressure; ALT, alanine aminotransferase; WC, waist circumference; TC, thigh circumference; CC, calf circumference

### Hazard ratios of malignancy-related mortality categorized by the different anthropometric indices

Table 7 demonstrates the HRs of malignancy-related mortality in the subjects with different anthropometric indices. The HRs and 95% CIs of the highest quartile compared with the base quartile for individuals with TC, CC, TC/WC, CC/WC and (TC + CC/WC) domains in the unadjusted model were 0.277 (95% CI: 0.158 to 0.485), 0.330 (95% CI: 0.193 to 0.565), 0.123 (95% CI: 0.064 to 0.239), 0.112 (95% CI: 0.056 to 0.225), and 0.121 (95% CI: 0.062 to 0.234), respectively (all *P* < 0.001). The HRs and 95% CIs of the highest quartile compared with the base quartile in the totally adjusted model in the different anthropometric indices listed above were 0.464 (95% CI: 0.185 to 1.168; *P* = 0.103), 0.310 (95% CI: 0.139 to 0.691; *P* = 0.004), 0.618 (95% CI: 0.284 to 1.346; *P* = 0.226), 0313 (95% CI: 0.142 to 0.688; *P* = 0.004) and 0.486 (95% CI: 0.224 to 1.055; *P* = 0.068), respectively. These finding supported that a higher CC/WC level had an inverse correlation with the risk of death from malignancy-related mortality in all quartiles.

**Table 7:** HRs of malignancy-related mortality categorized by the anthropometric parameters

Anthropometric Parameters	Quartiles	Unadjusted Model HR (95% CI)	*P* Value	Total Adjusted Model HR (95% CI)	*P* Value
TC	Q2 v.s. Q1 Q3 v.s. Q1 Q4 v.s. Q1	0.607 (0.396, 0.930) 0.303 (0.178, 0.518) 0.277 (0.158, 0.485)	0.022 <0.001 <0.001	0.775 (0.479, 1.253) 0.412 (0.212, 0.799) 0.464 (0.185, 1.168)	0.298 0.009 0.103
CC	Q2 v.s. Q1 Q3 v.s. Q1 Q4 v.s. Q1	0.657 (0.429, 1.007) 0.305 (0.176, 0.529) 0.330 (0.193, 0.565)	0.054 <0.001 <0.001	0.631 (0.397, 1.001) 0.271 (0.143, 0.513) 0.310 (0.139, 0.691)	0.051 <0.001 0.004
TC/WC	Q2 v.s. Q1 Q3 v.s. Q1 Q4 v.s. Q1	0.324 (0.206, 0.512) 0.190 (0.109, 0.331) 0.123 (0.064, 0.239)	<0.001 <0.001 <0.001	0.569 (0.353, 0.916) 0.574 (0.309, 1.067) 0.618 (0.284, 1.346)	0.020 0.079 0.226
CC/WC	Q2 v.s. Q1 Q3 v.s. Q1 Q4 v.s. Q1	0.280 (0.172, 0.456) 0.271 (0.166, 0.441) 0.112 (0.056, 0.225)	<0.001 <0.001 <0.001	0.332 (0.201, 0.549) 0.518 (0.302, 0.887) 0.313 (0.142, 0.688)	<0.001 0.017 0.004
(TC + CC)/WC	Q2 v.s. Q1 Q3 v.s. Q1 Q4 v.s. Q1	0.297 (0.185, 0.475) 0.198 (0.115, 0.341) 0.121 (0.062, 0.234)	<0.001 <0.001 <0.001	0.469 (0.287, 0.765) 0.532 (0.292, 0.971) 0.486 (0.224, 1.055)	0.002 0.040 0.068

Adjustment for age, sex, race/ethnicity, BMI, SBP, serum fasting glucose, serum cholesterol, serum albumin, ALT, serum uric acid, C-reactive protein, history of congestive heart failure, coronary heart disease, angina/angina pectoris, heart attack, stroke, cancer/malignancy, smoking, moderate to vigorous recreational activity

Abbreviation: BMI, body mass index; SBP, systolic blood pressure; ALT, alanine aminotransferase; WC, waist circumference; TC, thigh circumference; CC, calf circumference

## DISCUSSION

In the primary outcomes of present study, the most prominent finding was a positive correlation between the telomere length and the specific anthropometric parameters (TC and CC) in the highest quartile in all models. It is tempting to speculate that people with higher TC and CC values tend to have a longer relative telomere length due to the significant p value trends in our study. To the best of our knowledge, our study is the first to indicate the positive correlation between the telomere length and specific anthropometric parameters (TC and CC). In the secondary outcomes of our study, we observed a significant inverse association between the TC and CC and all-cause mortality in the longitudinal follow-up even after adjusting for all confounding factors, which lowered the hazards for all-cause mortality to 43% and 57%, respectively. A previous study demonstrated that the WC was inversely associated with the telomere length [11], consisted with the results of our study.

We investigated the most proper anthropometric index to serve as a clinical predictor for the reduction of the mortality risk. Therefore, we combined different anthropometric parameters that we observed to have significant relationships in our study, including positive and inverse relationships with telomere length. We put the anthropometric parameters (CC and TC) that had positive associations with telomere length as a numerator; and put the WC which had an inverse association with telomere length as a denominator. Therefore, higher values of the anthropometric indices we designed, might represent a relatively longer telomere length in the subjects. For all-cause mortality, all of the anthropometric indices investigated in our study that had higher values significantly lowered the hazard (especially CC, which showed the lowest hazard ratio). For cardiovascular mortality, higher CC and CC/WC levels were significantly associated with lower mortality in all quartiles. Furthermore, a higher CC/WC level had an inverse correlation with the risk of death from malignancy-related mortality. To summarize, the anthropometric indices involving the CC with higher values seemed to be surrogate markers for the reduction of the risk of all-cause, cardiovascular disease and malignancy-related mortality (especially CC/WC, which displayed a broad predictive ability according to the results of our study).

Although the factors involved in telomere length maintenance are incompletely understood, one possible mechanism directly linking a positive correlation between the lower extremity circumference and telomere length may be attributed to physical exercise. Generally, the telomere length is influenced by the activity of telomerase, which is the reverse transcriptase that maintains telomeres [20]. Werner C et al reported that voluntary physical exercise activated telomere-stabilizing proteins and telomerase activity to induce anti-senescent and protective effects [21]. Another article published in 2009 also exhibited that physical activity activated telomere-stabilizing proteins and thereby provided protection against vascular apoptosis [22]. The paper in LaRocca proposed that telomere length was preserved in healthy older adults who performed vigorous aerobic exercise [23]. Furthermore, Chilton and his colleagues observed that intense cardiorespiratory exercise was adequate to differentially activated key telomeric genes that might contribute to telomere homeostasis [24]. These results may offer one suitable explanation for why the lower extremity circumferences were positively correlated with telomere length: adequate physical exercise could result in skeletal muscle hypertrophy and increased muscular strength.

Several previous studies highlighted the relationship between different anthropometric indices and mortality in western populations. From the study of the Canada Fitness Survey, which enrolled 10,638 adults aged 20 to 69 years, reported that waist and extremity circumferences appeared to have opposite and independent effects on mortality in adults [25]. Reis proposed that the ratio measurements of the fat distribution, such as the waist-to-thigh ratio (WTR) and waist-to-hip ratio (WHR), had strong positive associations with death and provided extra prognostic information that was not provided by the WC and BMI in middle-aged populations from the NHANES III study [26]. A low TC was associated with an increased risk of premature death and hypothesized a common threshold of 60 centimeters [27]. Information concerning the relationship between the CC and mortality is relatively limited. However, Tsai and his colleagues found that the CC had a better predictive efficacy concerning the risk of long-range mortality than the BMI in elderly subjects in Taiwan, who were mostly confined to the Asian population [28]. Other research articles indicated potential roles for the CC in the association with health status. Bonnefoy M et al demonstrated that the CC was a pertinent marker of the nutritional state with the cutoff of 30.5 centimeters to provide a high-quality diagnostic capacity [29]. A cross-sectional study of the Aging and Longevity Study in the Sirente geographic area reported that a higher CC might be positively related to a higher functional performance and lower frailty index [30]. The paper by Hsu et al showed that the CC could be applied to predict emerging care needs in older adults [31]. Moreover, the CC was significantly associated with exercise intolerance and the functional capacity in patients with chronic lung disease [32].

Generally, the extremities have a relatively lower fat mass than other body sites. In a study concerning the reliability of standard circumferences in domain-related constitutional applications, the CC was strongly connected with the muscle mass rather than the fat mass at the segmental and total levels [33]. An earlier study conducted by Rolland, noted that the CC was positively associated with the dual-energy x-ray absorptiometry (DEXA)-measured appendicular skeletal muscle mass (ASM) in elderly subjects aged at least 70 years [34]. In an observational study of 3659 participants aged 55 years or more, the muscle mass index could perform as a predictor of longevity in older adults [35]. Additionally, the condition of losing muscle mass plus low physical performance or low muscle strength (which was named sarcopenia) showed an increase in mortality risk in the elderly [36]. In a longitudinal study of 345 elderly in Mexico City during the 3-year follow-up, the CC had a positive relationship with the ASM and could be applied as a surrogate marker for the muscle mass to diagnose sarcopenia in older Japanese adults [37]. These study results might indicate potential mechanisms to explain the connection between the CC and all-cause mortality in terms of deterioration of the fat-free or total muscle mass. Furthermore, multiple chronic medical diseases that might contribute to an increased risk of mortality, including type 2 diabetes mellitus, chronic obstructive pulmonary disease, stroke and coronary heart disease, were also correlated with a lower muscle mass according to the result of a 27-year follow-up prospective cohort study [38].

In our study, higher CC and CC/WC levels were significantly associated with lower cardiovascular mortality. These findings were in line with Takagi's research, which CC measurements might be applied to the prevention of cardiovascular events [39]. Park and his colleagues reported that the CC was correlated with carotid atherosclerosis in Korean patients with type 2 diabetes [40]. Furthermore, a low CC and high WC were observed to have a positive correlation with an increased risk of carotid atherosclerosis in Korean diabetic patients, and the waist-to-calf ratio was independently associated with a carotid atherosclerotic burden [41]. A prospective study of 6265 subjects aged more than 65 years in three French cities revealed that an inverse relationship between carotid plaques and the CC, with a higher CC having an anti-atherogenic effect in elderly subjects [42]. The probable interactions between cardiovascular disease and the CC described above provided hints to explain our study results that a higher CC reduced the risk of cardiovascular mortality.

The potential pathophysiology for anthropometric indices, including the CC and CC/WC, which presented an inverse association with malignancy-related mortality in our study, might contribute to skeletal muscle wasting and cachexia. Cachexia is a multi-factorial syndrome characterized by a constant run off of the skeletal muscle mass that cannot be fully recovered by a conservative nutritional supply and leads to further functional impairment [43]. Tsai S proposed that a low lean body mass was a predictor of adverse events among patients with cancer [44]. Several studies demonstrated that deletion of the skeletal muscle mass was a major determinant of a poor prognosis for cancer survival and limited the therapeutic efficacy of medical treatments, including palliative chemotherapy [45], neoadjuvant chemotherapy [46], and surgical resection, in many types cancer [47]. A previous study proposed probable mechanisms of cancer-related muscle loss associated with inflammation, which induced excessive fatty acid oxidation to cause muscle atrophy [48]. These findings suggest that early detection of a low muscle mass level may help identify subjects with cancer who are most in need of intervention or further evaluation to reduce the risk of mortality.

Numerous potential limitations need to be considered in this study. First, this study is an observational research study; therefore, we are unable to draw causal inferences between anthropometric parameters and telomere length and even cause-specific mortality because the single measurement of anthropometric parameter levels during the follow-up period may produce biased results. Thus, further studies are warranted to verify whether changes in telomere length can be assessed based on the CC or TC. Second, the subjects in our study with higher CC or TC values often also had a higher BMI, which was regarded as a risk factor for telomere length shortening [19]. However, a BMI cannot provide an explanation for the weight contributions made by different organs or lean and fat tissues or present the exact body fat distribution. Third, the anthropometric indices in our study included two or three parameters with ratios, such as CC/WC. For instance, the WC is a symbol of variability in intra-abdominal and subcutaneous fat, whereas the CC symbolizes changeability in subcutaneous fat and muscle. The combination of the CC with the WC may offer specific predictive information regarding extremely diverse but significant body sites. Therefore, ratio measurements may be queried to perform complex interpretations. An increased CC/WC may reflect a relative abundance of peripheral muscle (high CC), decreased visceral fat (low WC), or both. However, there is no reason to believe that the results in our study will be influenced by the controversy due to the common positive effects on telomere length and survival.

In summary, this study illustrated a positive correlation between telomere length and specific anthropometric parameters (TC and CC) with data from a nationally representative cohort. Additionally, the anthropometric indices involving CC with higher values seemed to be surrogate markers for a reduced risk of all cause, cardiovascular and malignancy-related mortality (especially the calf-to-waist ratio, which displayed a broad predictive ability). Thus, CC may be a valuable tool to guide public health policy and a clinical prognostic indicator for the risk of mortality. Further longitudinal studies concerning the benefits of the prevention of CC decline and the improvement of lower leg muscles through exercise training are warranted in clinical practice.

## MATERIALS AND METHODS

### Ethics statement

This study was exempt from Institutional Review Board (IRB) review because we investigated de-identified information from NHANES database, which had been approved by National Center for Health Statistics (NCHS) IRB.

### Data source and participants

The data are derived from the National Health and Nutrition Examination Survey (NHANES) from 1999 to 2002. The survey used a stratified multistage probability design with planned oversampling of certain age and minority groups and was a nationally representative health survey of non-institutionalized U.S. citizens recruited as participants. The study was conducted by the NCHS of the Centers for Disease Control and Prevention (CDC). It consisted of an in-home personal interview and standard physical examinations performed at mobile centers in accordance with the Declaration of Helsinki. Detailed consent documents and survey operation manuals for the 1999 to 2002 NHANES are available from the NHANES website.

### Measurement: telomere length

Blood samples were acquired from all subjects at least 20 years of age who were requested to provide DNA samples during the NHANES 1999 to 2000 or 2001 to 2002 surveys. A small portion of the samples was extracted and stored at −80°C to perform further DNA analysis. The telomere length analysis was executed with the quantitative polymerase chain reaction (PCR) method to quantify the telomere length compared with standard reference DNA (T/S ratio) in the outside laboratory of Dr. Elizabeth Blackburn at the University of California, San Francisco, as previously comprehensively described [49]. Each sample was assayed in duplicate wells, resulting in six data points for three time points on three different days. The sample plates were analyzed in groups of three plates, and each plate was not clustered more than once. Any plate included ninety-six controlled wells with eight controlled DNA models. Assays run at least eight times with invalid controlled wells were excluded (> 99% of the runs fitted this principle). Additionally, the mean T/S ratio value was calculated without possible outliers that symbolized the highest and lowest T/S ratio values in the set.

### Measurement: anthropometric parameters

All standard protocols to evaluate anthropometric parameters in our study are accessible on the NHANES website. We obtained the BMI according to the formula that divided the subject's weight in kilograms by the square of the height in meters (kg/m2). The weight was measured in pounds and converted to kilograms in the automated system with the participants wore only underwear, stood in the center of the scale platform and looked straight ahead. The height was measured in the standing position with the arms and shoulders relaxed to obtain maximum vertical size. The participants stood on the standiometer which consisted of a moveable headboard and a vertical backboard, with the heels of both feet together to ensure the body weight was distributed and both feet were flat on the floor.

The AC was measured in the standing position with relaxed shoulders, with the right arm hung droopily to ensure that the muscle of the arm did not exhibit flexion or tightening. Trained NHANES staff placed the measuring tape around the upper arm at the middle point between the acromion process of the scapula and the olecranon process vertical to the long axis of the upper limb and recorded the value to the nearest 0.1 centimeter.

The CC was measured in a sitting position. The measuring tape was placed around the right calf to find the position of the highest circumference vertical to the long axis of the lower leg to the nearest 0.1 centimeter.

To measure the WC, trained NHANES staff first had to locate the bony landmark of the lateral border of the ilium. Operators palpated the subject's hip region to localize the right ilium, drew a horizontal line just on top of the upmost landmark and placed the measuring tape around the trunk in a horizontal plane. The measurement was performed in the phase of the ending of the common expiration and was recorded to the nearest 1.0 millimeter.

To measure the TC, the operators asked the participants to stand with the right leg forward, the knees slightly bent and the soles of both feet planted on the floor to ensure that nearly all of the body weight was distributed onto the left leg. Trained study staff put the measuring tape around the middle part of the thigh in a plane vertical to the long axis of the thigh and measured to the nearest millimeter.

### Measurement: outcomes

The telomere length is an important factor in maintaining the health status and has been closely related to cardiovascular disease in earlier articles [5]. Furthermore, emerging studies indicated a positive connection between mortality and a shorter telomere length in recent [8–10]. Given that the association between telomere length and all-cause mortality, different anthropometric indices which had a significant association with telomere length might have potential correlation with mortality risk. Base on the abovementioned correlations, we further speculated a possible relationship existence that the subjects with higher CC or TC had a longer telomere length and lower mortality risk. Thus, we clarified the relationship between the anthropometric parameters or indices and mortality, including of all-cause, cardiovascular and malignancy-related deaths. The follow-up data on all-cause, cardiovascular and malignancy-related mortality were obtained from the NHANES study, which contained detailed information about mortality from the time of study subjects’ examination dates to death or December 31, 2006. The data were provided by NCHS based on probabilistic matching in terms of the National Death Index death certificate records [50]. Informed consent was ignored because this study was a type of retrospective cohort study with minimal privacy risks and human subject contact.

### Measurement: covariates

The associated information concerning variables, such as age, gender, race/ethnicity, smoking status, and recreational activity, and medical conditions diagnosed by a doctor, including heart attack, angina/angina pectoris, coronary heart disease, congestive heart failure, stroke and cancer/malignancy, were collected by self-report. The definition of recreational activity in our study was limited to the moderate to vigorous degree that induced an elevation in heart rate or breathing frequency, such as bicycling, swimming or running for at least 10 minutes without interruption.

Serum plasma glucose was measured from blood samples derived from participants who had fasted for at least six hours. The biochemical analysis of the plasma glucose was measured by the hexokinase enzymatic method according to the Cobas Mira Chemistry System (Roche Diagnostic Systems, Indianapolis, IN, USA) by the Department of Child Health at the University of Missouri-Columbia. Serum total cholesterol was measured with the Hitachi 704 Analyzer (Roche Diagnostics, Indianapolis, IN, USA) by the Lipoprotein Analytical Laboratory at Johns Hopkins University School of Medicine. The serum C-reactive protein (CRP) level was obtained by latex-enhanced nephelometry (Behring Nephelometer II Analyzer System; Behring Diagnostics Inc., Somerville, NJ, USA). The other biochemical profiles, including serum uric acid, serum albumin and alanine aminotransferase (ALT), were measured using the Beckman Synchron LX20 instrument. All of the protocols used standardized principles with documented accuracy in the CDC reference methods.

### Statistical analysis

All data analyses were performed with the Predictive Analytics Suite Workstation Statistics, version 18.0 for Windows (SPSS Inc., Chicago, IL, USA), which incorporated sampling weights and prevented incorrect estimates of variance by the Complex Samples procedure. We investigated the association between anthropometric parameters, including AC, WC, TC and CC, with telomere length using multivariable logistic regression models designed with progressive degrees of modification. An extended-model approach was executed for covariate adjustment as follows: Model 1 was not adjusted for other variables; Model 2 was adjusted for age, gender, race, BMI, systolic blood pressure, serum fasting glucose, serum total cholesterol, serum albumin, ALT, serum uric acid, and CRP; and Model 3 = Model 2 + medical history of heart attack, angina/angina pectoris, coronary heart disease, congestive heart failure, stroke, cancer/malignancy, smoking, and moderate to vigorous recreational activity.

Categorical variables were summarized by their means and standard deviations (SD), whereas continuous variables were summarized by their frequency counts and percentages. The Chi-square test was used with categorical data and the Wilcoxon Rank sum test was used with continuous data. We examined the TC from 7,507 participants with quartiles and the CC in 7,629 participants with quartiles to distinguish the demographic characteristics without specific limitations in gender and ethnicity. A p value less than 0.05 was regarded as statistically significant (2-sided).

The relationship between the anthropometric parameters or indices in subjects and the all-cause, cardiovascular and malignancy-related mortality was investigated with Kaplan-Meier survival curves. Multivariate Cox proportional hazard ratio models classified into unadjusted and total adjustment as mentioned above (model 3) were applied to assess the connections between the quartiles of different anthropometric indices and all-cause, cardiovascular and cancer mortality. The anthropometric indices in our study included TC/WC, CC/WC and (TC + CC/WC). For example, TC/WC symbolized the thigh circumference divided by the waist circumference. These indices allowed us to use combinations of different anthropometric parameters to evaluate the potential efficacy and present the true muscle mass distribution in all subjects.
